# Meta-Analysis of Gadoxetic Acid Disodium (Gd-EOB-DTPA)-Enhanced Magnetic Resonance Imaging for the Detection of Liver Metastases

**DOI:** 10.1371/journal.pone.0048681

**Published:** 2012-11-07

**Authors:** Lihua Chen, Jiuquan Zhang, Lin Zhang, Jing Bao, Chen Liu, Yunbao Xia, Xuequan Huang, Jian Wang

**Affiliations:** 1 Department of Radiology, Southwest Hospital, Third Military Medical University, Chongqing, China; 2 Department of Radiology, Taihu Hospital, Wuxi, Jiangsu Province, China; 3 Department of Pathogen Biology, School of Medicine, Nantong University, Nantong, Jiangsu Province, China; NIH, United States of America

## Abstract

**Objective:**

To determine the accuracy of MR imaging with Gd-EOB-DTPA for the detection of liver metastases.

**Materials and Methods:**

PUBMED, EMBASE, the Web of Science, and the Cochrane Library were searched for original articles published prior to February 2012. The criteria for the inclusion of articles were as follows: reported in the English language; MR imaging with Gd-EOB-DTPA was performed to detect liver metastases; histopathologic analysis (surgery, biopsy), intraoperative observation (manual palpatation, intraoperative ultrasonography), and/or follow-up US was the reference standard; and data were sufficient for the calculation of true-positive or false-negative values. The methodological quality was assessed by using the quality assessment of diagnostic studies instrument. The data were extracted to calculate sensitivity, specificity, predictive value, diagnostic odds ratio, and areas under hierarchical summary receiver operating characteristic (HSROC) curve to perform heterogeneity test and threshold effect test, as well as publication bias analysis and subgroup analyses.

**Results:**

From 229 citations, 13 were included in the meta-analysis with a total of 1900 lesions. We detected heterogeneity between studies and evidence of publication bias. The methodological quality was moderate. The pooled weighted sensitivity with a corresponding 95% confidence interval (CI) was 0.93 (95% CI: 0.90, 0. 95), the specificity was 0.95 (95% CI: 0.91, 0.97), the positive likelihood ratio was 18.07 (95% CI: 10.52, 31.04), the negative likelihood ratio was 0.07 (95% CI: 0.05, 0.10), and the diagnostic odds ratio was 249.81 (95% CI: 125.12, 498.74). The area under the receiver operator characteristic curve was 0.98 (95% CI: 0.96, 0.99).

**Conclusion:**

MR imaging with Gd-EOB-DTPA is a reliable, non-invasive, and no-radiation-exposure imaging modality with a high sensitivity and specificity for detection of liver metastases. Nonetheless, it should be applied cautiously, and large scale, well-designed trials are necessary to assess its clinical value.

## Introduction

The liver is one of the most common sites for metastatic disease, accounting for 25% of all metastases to solid organs, and secondary liver neoplasms are far more common than primary hepatic neoplasms [1]. The accurate detection of liver metastases is crucial for determining treatment planning and for improved therapeutic outcomes. Both interventional therapies and complete resection of hepatic metastases have been shown to increase survival in patients with colorectal cancer and other selected malignancies [2], [3]. Therefore, exact number and location of hepatic metastases is essential for the success of these therapies.

Gadolinium ethoxybenzyl diethylenetriamine pentaacetic acid (gadoxetic acid disodium or Gd-EOB-DTPA, Primovist, Schering, Berlin, Germany) can be used effectively in dynamic and liver-specific magnetic resonance (MR) imaging, and it has several advantageous properties for evaluating liver lesions. About 20 minutes after injection, normal areas of the liver exhibit T1 shortening because of specific hepatocyte uptake of approximately 50% of the injected dose, whereas hepatic metastases, do not exhibit T1 shortening [4]–[6]. Combined with the three-dimensional gradient-echo sequence technique, which provides excellent spatial resolution, gadoxetate disodium–enhanced MR imaging may be useful in detecting small liver lesions [7].

Although a large number of studies have reported the superior value of dynamic contrast enhanced MR imaging with the use of Gd-EOB-DTPA for assessment of liver metastases, the findings have been largely incongruent and the sample size in these studies was relatively small. Otherwise, evidence of evidence-based medicine with the use of Gd-EOB-DTPA is almost zero. We designed a meta-analysis to evaluate the published experimental data regarding MR imaging with the use of Gd-EOB-DTPA for the detection of liver metastases to determine diagnostic value of this imaging method and provide evidence of evidence-based medicine for clinical diagnosis.

**Figure 1:**
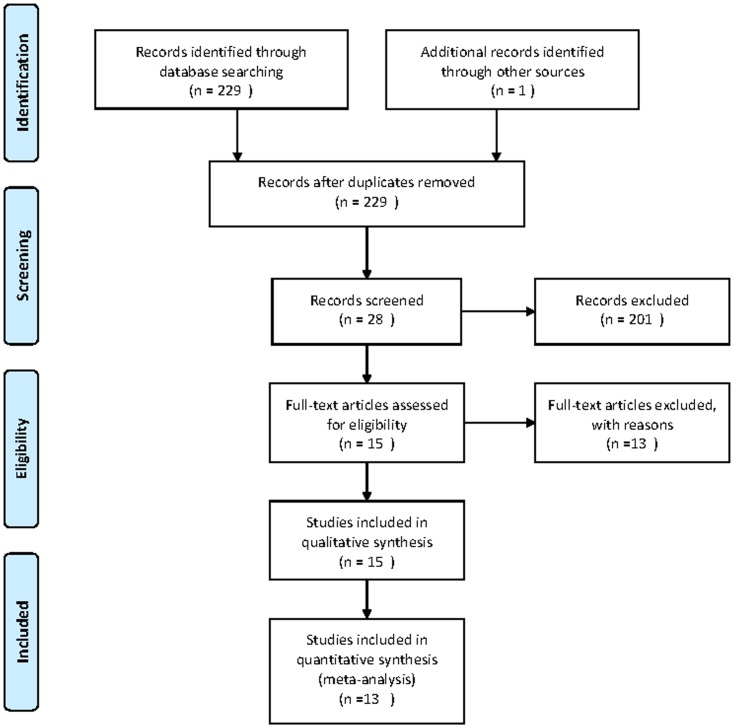
Flowchart illustrating the selection of studies.

**Table 1:** Characteristics of the included studies.

Study	Year	TP	FP	FN	TN	Patient	Nation	Patient Spectrum*	Study design	blind	Method
Schreiter [16]	2012	99	4	14	26	143	Germany	liver metastases	prospective	Y	1.5T
Goshima [17]	2010	28	4	1	28	61	Japan	liver metastases	retrospective	Y	3.0T
Kim [18]	2010	75	2	6	16	99	Korea	liver metastases	retrospective	Y	1.5T
Lee, M [19]	2011	78	2	1	37	118	Korea	liver metastases	retrospective	Y	3.0T
Shimada [20]	2010	48	2	4	52	106	Japan	liver metastases	retrospective	Y	3.0T
Chung [23]	2011	77	5	2	29	113	Korea	CRLM	retrospective	Y	3.0T
Sofue [22]	2011	81	5	7	51	144	Japan	CRLM	retrospective	Y	3.0T
Kulemann [21]	2011	45	0	6	13	64	Austria	CRLM	retrospective	Y	1.5/3.0T
Muhi, A [24]	2011	56	1	3	206	266	Japan	liver metastases	retrospective	Y	1.5T
Motosugi [25]	2011	48	3	10	45	106	Japan	CRLM	retrospective	Y	1.5T
Lowenthal [26]	2010	268	2	10	52	332	Germany	liver metastases	retrospective	Y	1.5T
Donati [29]	2010	50	0	5	30	85	Switzerland	CRLM	retrospective	Y	1.5T
Seo [30]	2011	123	2	12	28	165	Korea	CRLM	retrospective	Y	3.0T

* CRLM  =  colorectal liver metastases.

**Figure 2:**
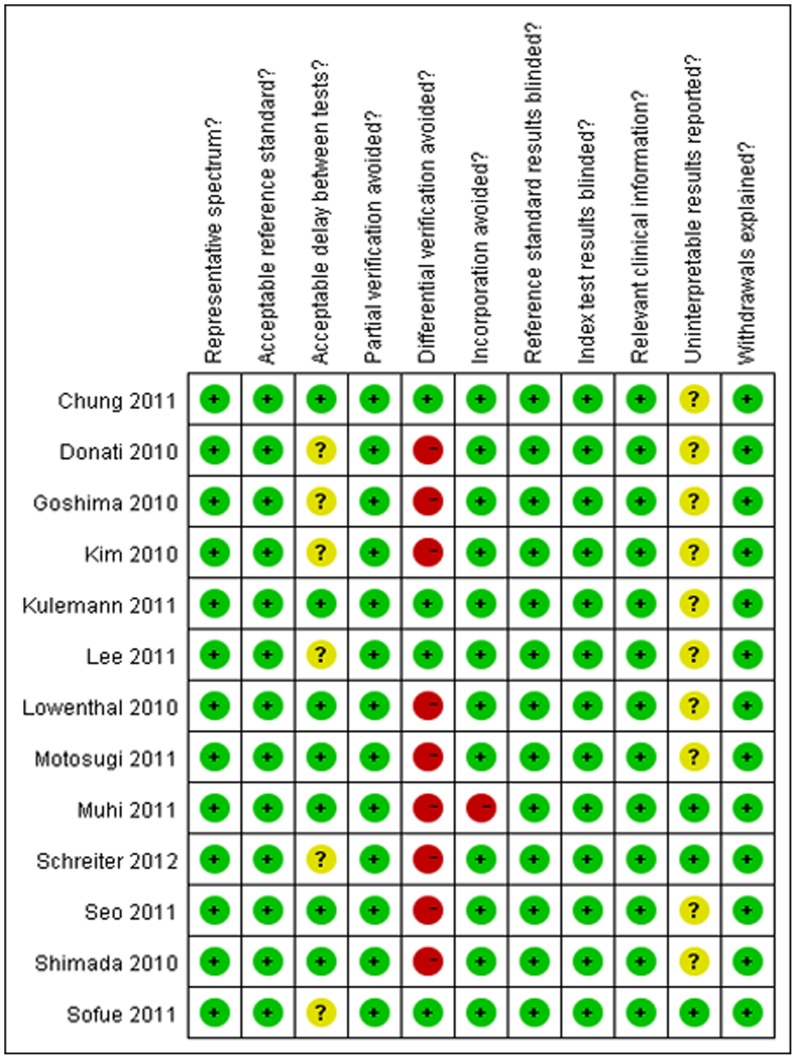
Methodological quality of the 13 included studies on a per-lesion basis.

## Materials and Methods

### Literature Search

The PUBMED, EMBASE, the Web of Science, and the Cochrane Library were searched independently by two observers using the terms “Gadolinium-EOB-DTPA OR gadoxetic acid disodium OR Gd-EOB-DTPA OR eovist OR primovist” for the diagnostic test and “Liver metastases OR hepatic metastases OR liver lesions” for the clinical domain. The search strategy was based on the Bayes Library of Diagnostic Study and Reviews. We limited our search to publications in the English language with the presence of the search term in the title or abstract of the article and a publication date no later than February 2012. Review articles, letters, comments, case reports, and unpublished articles were excluded. Extensive crosschecking of the reference lists of all retrieved articles was performed.

**Figure 3:**
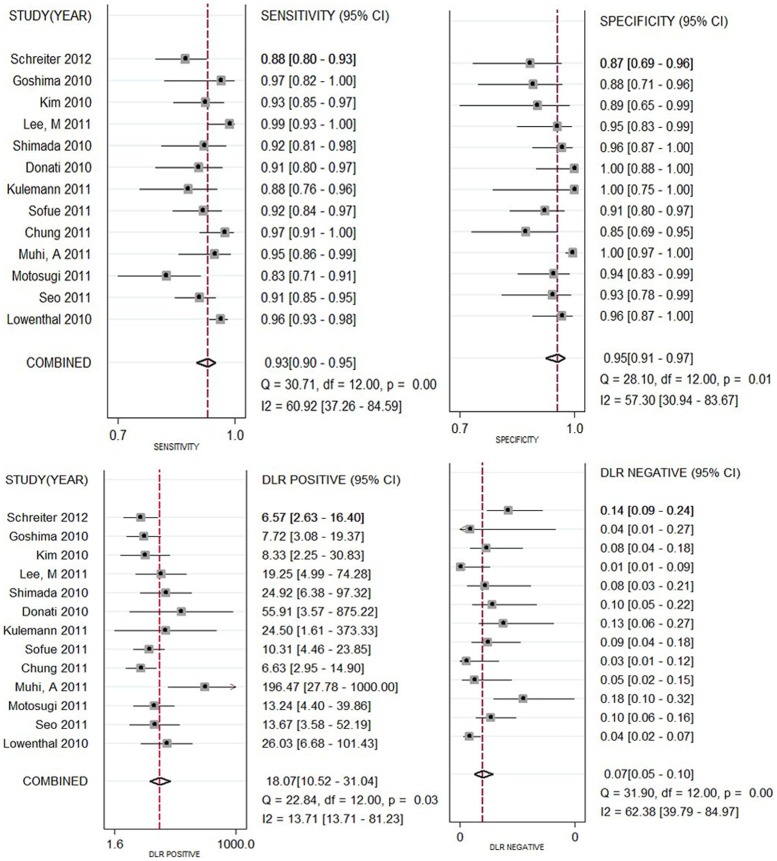
Forest plots of the SEN, SPE, PLR and NLR with corresponding 95%CIs for MR imaging with Gd-EOB-DTPA for the detection of liver metastases.

**Figure 4:**
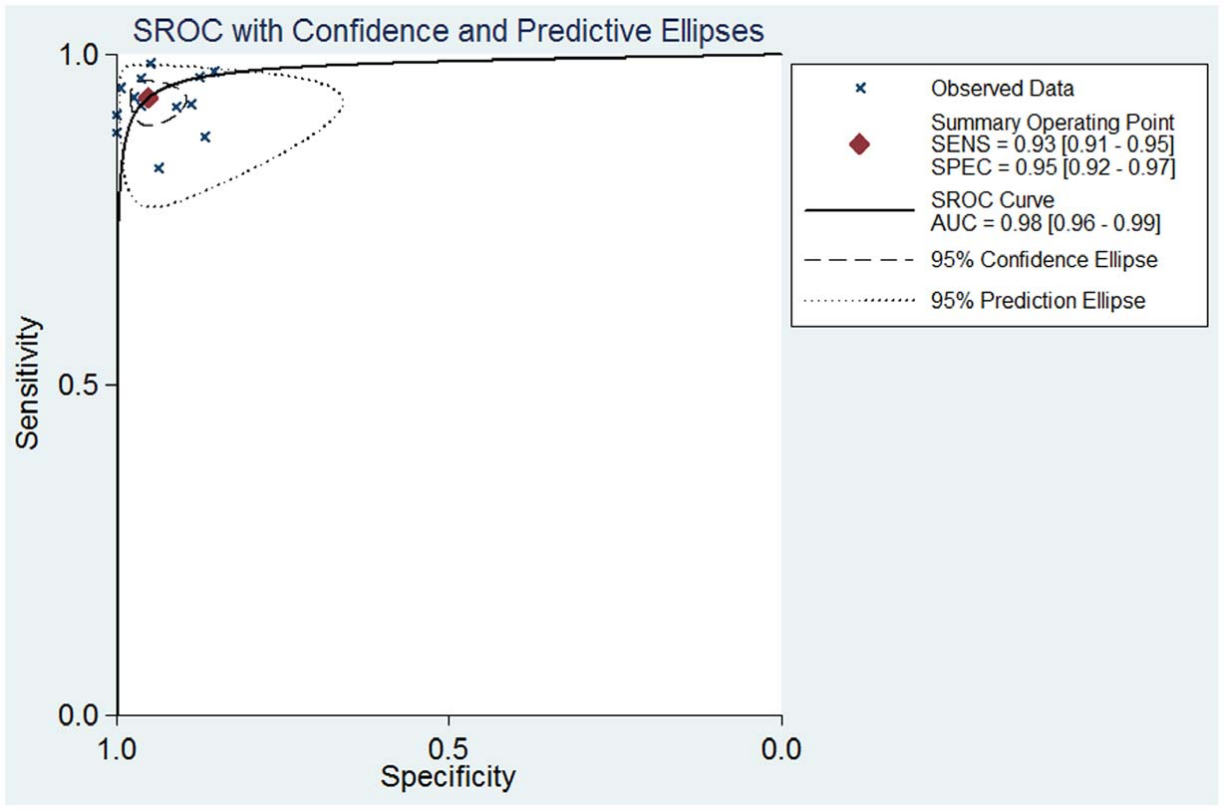
Summary receiver operating characteristic (SROC) curves from the bivariate model of MR imaging with Gd-EOB-DTPA in detection of liver metastases.

### Selection of Articles

The articles selected for inclusion met the following criteria: the articles were reported in the English language; MR imaging with Gd-EOB-DTPA was performed to identify and characterise liver metastases; histopathologic analysis (surgery, biopsy), intraoperative observation (manual palpatation, intraoperative ultrasonography), and/or follow-up US or CT was the reference standard; and the data were sufficient for the calculation of true-positive, false-positive(), true-negative or false-negative values. Studies were excluded if (a) there were fewer than 20 patients; (b) multiple reports were published for the same study population (in this case, the publication with the most details and/or most recently published was chosen); and (c) the study included patients who had previously undergone treatment for liver metastases.

**Table 2:** Sensitivity estimates for each subgroup on a per-lesion basis.

Subgroup	No. of Studies	Mean Sensitivity (%)	P Value
Lesion size			
<10 mm	6	79(72–85)	0.437
≥10 mm	6	97(95–99)	0.323
<10 mm vs. ≥10 mm	NA	NA	0.011
magnetic field strength			
1.5T	7	94(91–95)	0.077
3.0T	7	93(90–94)	0.001
1.5T vs. 3.0T	NA	NA	0.879
Study design			
retrospective	12	94(92–95)	0.001
prospective	1	87	NA
retrospective vs. prospective	NA	NA	0.082
Patient Spectrum			
CRLM	6	94(92–96)	0.071
liver metastases	7	91(88–94)	0.014
CRLM vs. liver metastases	NA	NA	0.289

Note.–Numbers in parentheses are the 95% CIs. NA  =  not applicable. CRLM  =  colorectal liver metastases.

### Quality Assessment and Data Extraction

The methodological quality of the included studies was assessed independently by two observers using quality assessment of diagnostic studies (QUADAS) instrument, a quality assessment tool specifically developed for systematic reviews of diagnostic accuracy studies [8], [9]. Meanwhile, the relevant data were also extracted from each study, including author, study nation, publication year, description of study population, study design characteristics, magnetic field strength, type of coil used, pulse sequences, and descriptions of interpretations of the diagnostic tests. To resolve disagreement between the reviewers, a third reviewer assessed all of the involved items. The majority opinion was used for the analysis.

**Figure 5:**
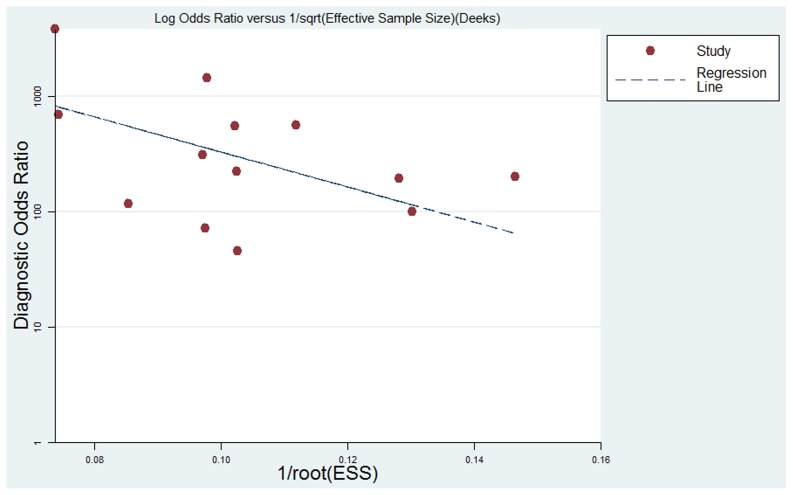
The funnel plot of publication bias. Linear regression of the inverse root of effective sample sizes (ESS) on a log dOR is performed as a test for funnel plot asymmetry.

For each study, values for true-positive (TP), false-positive, true-negative (TN), false-negative, sensitivity (SEN), specificity (SPE), positive likelihood ratio (PLR) and negative likelihood ratio (NLR) results for the detection of lesions were extracted, and 2×2 contingency tables were constructed.

### Meta-Analysis

Exploring heterogeneity is a critical issue to understand the possible factors influencing accuracy estimates and to evaluate the appropriateness of statistical pooling of accuracy estimates from various studies. The Q statistic of the Chi-square value test and the inconsistency index (I-squared, I^2^) were used to estimate the heterogeneity of the individual studies using the State software. P<0.1 or I^2^>50% suggested notable heterogeneity [10]. If notable heterogeneities were detected, the test performance was summarised by using a random-effects coefficient binary regression model; otherwise, a fixed-effects coefficient binary regression model was used [11].

In test accuracy studies, one of the primary causes of heterogeneity is the threshold effect, and arises when different cut-offs or thresholds are used in different studies to define a positive (or negative) test result. The Spearman correlation coefficient between the logit of sensitivity and the logit of (1-specificity) was computed to assess the threshold effect using Meta-Disc version 1.4. A strong positive correlation would suggest a threshold effect, P<0.05 [12], [13]. We constructed hierarchical summary receiver operating characteristic (HSROC) curves to assess SEN and SPE [14]. The areas under the ROC curves (AUC) were used to analyse the diagnostic precision of MR imaging with Gd-EOB-DTPA for the detection of liver metastases on a per-lesion basis.

Apart from variations due to the threshold effect, there are several other factors that can result in variations in accuracy estimates amongst different test accuracy studies in a review. In this study, meta-regression was used to explore such heterogeneity by relating the accuracy measurement to study level co-variates (such as, reference standard, study nation, publish year, study design, MRI field strength, or the location of primary tumor). Subgroup analyses were performed on a per-lesion basis. The following subgroups were created: (a) comparisons for the lesions smaller than 10 mm or those 10 mm or larger; (b) comparisons between studies with a prospective or retrospective design; (c) comparisons for the magnetic field strengths of 1.5T or 3.0T; (d) comparisons for lesions of liver metastases or colorectal liver metastases; (e) comparisons for liver metastases confirmed by histopathological examination only or by histopathological examination and else (intraoperative observation or follow-up).

The presence of publication bias was visually assessed by producing a Deeks funnel plot and an asymmetry test with the Stata software. Publication bias was considered to be present if there was a nonzero slope coefficient (P<0.05) [15].

## Results

The search initially yielded 229 potential literature citations through database searching and 1 additional record was identified through grey literature searching (Fig. 1). At the time of the review of the titles and abstracts, 201 of these studies were excluded because they were not relevant studies which used MR imaging with Gd-EOB-DTPA to detect liver metastases or they were not clinical trials. After reading the full texts, 13 of the remaining 28 articles were excluded because they lacked sufficient information to complete a 2×2 contingency table, the article was not found or the study was not published in English. After this final screening, 15 published studies–11 per-lesion [16]–[26], 2 per-patient [27], [28] and 2 per-lesion and per-patient [29], [30]–met our inclusion and exclusion criteria. As the sample size of the data reported on a per-patient basis were too small, the data analysis for these studies was performed only on a per-lesion basis (n = 13). The abstracted data of these individual studies are summarised in (Table 1). The quality assessment was moderate in 13 studies according to QUADAS items, and the results of the distribution of the study design are shown in Fig. 2.

As significant heterogeneity was found in the pooled analysis (I^2^ = 50.3%, P = 0.067), SEN, SPE, PLR, NLR were pooled by using a random-effects coefficient binary regression model. The pooled weighted values were determined to be SEN: 0.93 (95% confidence interval (CI): 0.90, 0. 95); SPE: 0.95 (95% CI: 0.91, 0.97); PLR: 18.07 (95% CI: 10.52, 31.04); NLR: 0.07 (95% CI: 0.05, 0.10); DOR: 249.81 (95% CI: 125.12, 498.74). The forest plots from 13 studies on a per-lesion basis are shown in Fig. 3. HSROC curves are shown in Fig. 4. The AUC was 0.98 (95% CI: 0.96, 0.99).

A Spearman rank correlation was performed as a further test for the threshold effect and was determined to be 0.149 (P = 0.628), which indicated that there was an absence of a notable threshold effect in the accuracy estimates among individual studies.

The results of meta-regression indicated that reference standard, study nation, publish year, study design, MRI field strength, or location of primary tumor were not strongly associated with accuracy. The sensitivity estimates for the different subgroups are presented in Table 2. The sensitivity of MR imaging with Gd-EOB-DTPA for lesions smaller than 10 mm was significantly lower than that for lesions measuring at least 10 mm (P = .001). For subgroups of reference standard, MRI field strength, study design and primary cancer sites, the results showed that they all had comparably high sensitivity estimates (P = 0.851, P = 0.879, P = 0.081, P = 0.289, respectively). The pooled weighted SEN of lesions smaller than 10 mm was 0.79 (95% CI: 0.72, 0.85), I^2^ = 0.0%, P = 0.437, and for lesions 10 mm or larger, the pooled weighted SEN was 0.97 (95% CI: 0.95, 0.99), I^2^ = 14.3%, P = 0.323.

The results of Deeks funnel plot asymmetry test (P = 0.084) showed evidence of notable publication bias (Fig. 5), which suggested that only small studies reporting high accuracy had been published, and small studies reporting lower accuracy had likely not been published.

## Discussion

To the best of our knowledge, this study is the first meta-analysis of the diagnostic performance of MR imaging with Gd-EOB-DTPA to assess liver metastases, although several previous meta-analyses and/or systematic reviews [31]–[34] have been performed to assess the imaging modality performance in the diagnosis of colorectal cancer liver metastases, which did not include data related to liver-specific gadolinium-containing contrast material, such as Gd-EOB-DTPA. According to Niekel *et al*. [34], MR imaging is the preferred first-line modality for evaluating colorectal liver metastases in patients who have not previously undergone therapy, and the pooled weighted sensitivity estimate on a per-lesion basis was 0.80 (95% CI: 0.75, 0.85). The pooled weighted sensitivity of our subgroups-analysis of MR imaging with Gd-EOB-DTPA for the detection of colorectal liver metastases was 0.94 (95% CI: 0.92, 0.96), which indicates excellent test performance. However, MR imaging with Gd-EOB-DTPA should be implemented cautiously because it is a relatively new technique–early publications concerning new techniques tend to be over-positive [35]–and the studies that have been published so far are more tend to have a retrospective design than a prospective design. So, this new technique should be implemented cautiously.

However, the homogeneity test of the sensitivity and specificity detected notable heterogeneity, which is consistent with previous meta-analyses and/or systematic reviews [31], [33], [34], [36]. Therefore, it is necessary to investigate the source of the heterogeneity.

We observed that the Spearman correlation coefficient was 0.149 (P = 0.628), which indicated that no significant threshold effect exists. To determine whether there are other sources of heterogeneity in addition to the threshold effect, a subgroup analysis is performed to detect factors that impact heterogeneity, such as study design, magnetic field strength, lesion size and patient spectrum (liver metastases or colorectal liver metastases). The results showed that the sensitivity for the detection of lesions measuring at least 10 mm was significantly higher than that of lesions smaller than 10 mm (P = 0.001), and other impact factors had comparably high sensitivity estimates (P = 0.879, P = 0.081, P = 0.289, respectively). Comparing with the pooled weighted SEN of the subgroups for lesion size, the ability of the imaging technique to detect the metastases of lesions larger than 10 mm differed from that for lesions smaller than 10 mm. Therefore, the content of lesions smaller than 10 mm did affect the diagnostic accuracy of study and may have contributed to the heterogeneity.

The study design may exhibit variation between studies in the selection of patients and in the test protocol, and this may be a source of bias and variability in meta-analysis [37]. Again, the effect of these differences will be estimated in the SROC, which may affect either sensitivity or specificity [38]. We constructed HSROC curves using a bivariate model, enabling an analysis of the effects on sensitivity and sensitivity separately [13], to assess the diagnostic performance. In this HSROC curve, the SEN, SPE and AUC were 0.93 (95% CI: 0.91, 0.95), 0.95 (95% CI: 0.92, 0.97), and 0.98 (95% CI: 0.96 0.99), respectively; these findings indicate an excellent diagnostic performance.

Our study design has some inherent limitations that should be considered when interpreting our results. First, we did not choose a per-patient basis analysis because the sample size of the data on a per-patient basis was too small. It is important to differentiate patients with liver metastases from those without liver metastases, although analysis on a per-patient basis most likely leads to overestimation of the sensitivity values. However, it is more important to perform data analyses on a per-lesion basis than that on a per-patient because the exact number, size and location of hepatic metastases are crucial requirements for therapy and information about the performance of a test can be obtained from the lesion data.

Second, there is notable publication bias in this study. Our meta-analysis was based only on published studies, which tend to report positive or significant results; the studies with nonsignificant or negative results are often rejected or are not even submitted. However, it is suggested that the quality of the data reported in articles accepted for publication in peer-reviewed journals is superior to the quality of unpublished data [39]. In addition, this review was restricted to articles published in English because other languages, such as Czech [40], would not be accessible, and this was also likely to introduce bias.

Third, the number of studies included in our meta-analyses is relatively small. However, a discussion of a systematic review [41] that studied the characteristics of meta-analyses and their included studies in the Cochrane Database showed that the number of studies eligible for meta-analysis is typically very small in all medical areas and for all outcomes and interventions covered by the Cochrane reviews. On the other hand, compared with the numbers of included studies, the methodological quality of the included studies has a more important influence on the estimated effects [42]. The quality assessment of our component studies according to QUADAS items was moderate.

### Conclusion

In conclusion, our meta-analysis demonstrated that MR imaging with Gd-EOB-DTPA is a reliable, non-invasive, and non-radiative imaging modality with high sensitivity and specificity for detection of liver metastases which previously undergone treatment. Nonetheless, as a relatively new technique, considering the notable heterogeneity and the existing inherent limitations, it should be applied cautiously, and large scale, well-designed trials are necessary to assess its clinical value.
